# Field evaluation of the P22 ELISA for diagnosis of caprine tuberculosis in an endemic area

**DOI:** 10.3389/fvets.2025.1628812

**Published:** 2025-07-24

**Authors:** Carlos Velasco, Javier Ortega, Julio Alvarez, Jose Antonio Infantes Lorenzo, José C. Moreno, Cristina Sanz, Beatriz Romero, Lucia de Juan, Lucas Dominguez, Mercedes Dominguez, Inmaculada Moreno, Alvaro Roy, Javier Bezos

**Affiliations:** ^1^VISAVET Health Surveillance Centre, Complutense University of Madrid, Madrid, Spain; ^2^Departamento de Sanidad Animal, Facultad de Veterinaria, Universidad Complutense de Madrid, Madrid, Spain; ^3^Reference and Research Laboratory for Respiratory Virus, Centro Nacional de Microbiología, Instituto de Salud Carlos III, Madrid, Spain; ^4^Servicio de Sanidad Animal, Dirección General de Agricultura y Ganadería, Mérida, Spain; ^5^Unidad de Inmunología Microbiana, Centro Nacional de Microbiología, Instituto de Salud Carlos III, Madrid, Spain; ^6^Centro Nacional de Epidemiología, Instituto de Salud Carlos III, Madrid, Spain

**Keywords:** goat, tuberculosis, intradermal tuberculin test, P22 ELISA, serum, milk, bulk tank milk

## Abstract

Animal tuberculosis (TB) affects a wide range of domestic species, including goats. TB eradication programs in goats are based on cell-based techniques such as the single and comparative intradermal tuberculin test (SITT and CITT, respectively). In recent years, an ELISA technique based on the P22 protein complex (P22 ELISA), has emerged as a valuable tool for TB diagnosis. The aim of the study was to evaluate the performance of the P22 ELISA in the context of a caprine TB eradication program using serum, individual milk and bulk tank milk (BTM) samples in order to define its usefulness in classifying herds compared to SITT and CITT. Samples from 53 herds categorized based on the detection of CITT reactors (16 high-risk herds, with one or more CITT reactors, and 37 low-risk herds, with only CITT-negative goats) were analyzed. Reactors in the P22 ELISA were detected in a higher number of high-risk herds using both serum (87.5%) and individual milk (81.3%) compared to SITT (75.0%) and CITT (31.3%), while the use of BTM led to the detection of 33.3% of the herds. Individual apparent prevalence was higher using the P22 ELISA in both serum (11.0%) and milk (15.0%) compared to the SITT (6.8%) and CITT (2.5%), with also a significantly (*p* < 0.001) higher number of reactors in individual milk compared with the serum. Similarly, all six herds with MTBC confirmed infection showed reactors to the SITT, CITT, and individual serum and milk P22 ELISA (2 out of 5 detected using BTM), although the highest reactivity was observed using individual milk samples. In the low-risk herds, a lower number of positive herds and animals were found with the P22 ELISA using serum or individual milk (51.4%) compared to SITT (59.5%) while using CITT only 2.7% of the herds were positive and none reacted to the P22 ELISA in BTM samples. This study shows that the P22 ELISA, using serum and especially individual milk samples, could be a complementary tool for maximizing the sensitivity of intradermal testing within the framework of a caprine TB eradication program.

## 1 Introduction

Animal tuberculosis (TB) is a chronic zoonotic disease caused by members of the *Mycobacterium tuberculosis* complex (MTBC), mainly *M. bovis* and *M. caprae* (1), that affects a wide range of domestic and wildlife species and humans (2, 3). Cattle have been traditionally considered the main domestic reservoir of TB infection (4). However, other species such as goats play an important role on the transmission and maintenance of infection (5–7) and are also responsible for cases of TB in humans (8). The animal and public health implications of TB coupled with the disease-associated economic losses highlight the importance of the diagnosis and control of caprine TB, especially in countries like Spain, which has the second largest population of goats in the European Union (2.3 million) (9) and in which the small ruminant sector represents nearly 10% of total livestock production (10). Nevertheless, goats are not subjected to compulsory TB eradication programs within the European Union, although the Spanish bovine TB eradication program includes surveillance and testing of caprine herds sharing pastures or epidemiologically linked to cattle herds (11). However, some regions have implemented voluntary or mandatory caprine TB eradication programs (12, 13). These programs are mainly based on test and cull strategies using the single or comparative intradermal tuberculin test (SITT or CITT, respectively), along with slaughterhouse surveillance (13–15). The limited sensitivity (Se) and specificity (Sp) of the intradermal tests in certain epidemiological settings (16–18) such as in flocks infected with (13, 19) or vaccinated against *Mycobacterium avium* subsp. *paratuberculosis* (MAP) (5, 20), highlights the need of additional diagnostic tests that can help overcome these limitations.

The P22 ELISA is an immunoassay that detects specific antibodies against P22, a protein complex immunopurified from bovine Purified Protein Derivative (PPD) (21–23). Despite the Sp of the P22 ELISA may be also compromised in scenarios of MAP infection or vaccination (18), it has demonstrated to be a useful technique to maximize Se of intradermal testing at small-scale studies (ranging from 1 to 3 herds per study) (21, 22, 24–26). Furthermore, serum P22 ELISA has been used in a large-scale epidemiological study assessing MTBC circulation in southern Spain (Andalusia), a high-prevalence caprine TB region of Spain (27). However, performance of the test using other type of samples such as individual milk samples or from the bulk tank (BTM) remains to be proven under field conditions at a larger scale. Milk samples may be particularly useful for TB screening in dairy caprine herds from regions or countries where compulsory testing of all animals is not affordable (24). Thus, the aim of the present study was to carry out the first large scale-study evaluating the performance of the P22 ELISA in individual serum and milk samples and BTM samples in goats in an endemic area of TB.

## 2 Materials and methods

### 2.1 Study design

**Figure 1:**
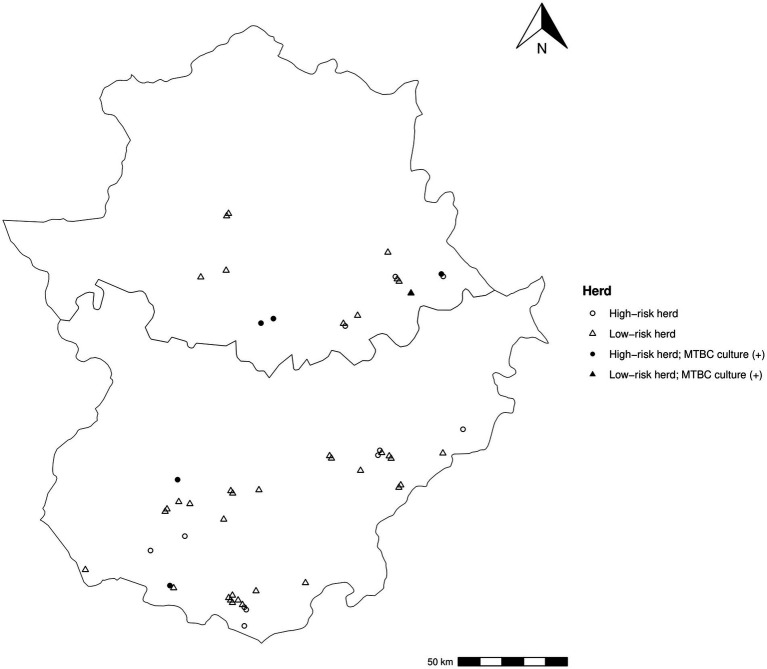
Distribution of the sampled herds in the region of Extremadura. Circles and triangles represent high and low-risk herds, respectively, and filled when MTBC culture confirmation.

Between 2018 and 2019, a cross-sectional study was performed in Extremadura (Spain), the third region in terms of goat population in Spain (10) and a high-prevalence region for caprine TB (6.0% herd-level prevalence based on CITT in 2017) (28) and bovine TB (9.7% herd-level prevalence based on SITT in 2017) (11). The study was performed in the framework of the current mandatory caprine TB eradication program of Extremadura (28). Fifty-three herds were randomly included in the study and classified into two groups according to their epidemiological history of TB in the previous routinary herd-testing: high risk herds (those with one or more CITT reactors, *n* = 16) and low-risk herds (those with only CITT-negative goats, *n* = 37) (Figure 1). No information regarding MAP infection or MAP-vaccination on these herds was available. The number of animals sampled in each herd was set to detect the presence of the TB-infection at a minimum estimated prevalence of 5.0% with a confidence level of 95% based on the size of each flock (Supplementary Table 1). A total of 2,129 goats (771 from high-risk herds and 1,358 from low-risk herds, Table 1) were finally enrolled in the study. The animals were subjected to CITT according to the regional caprine TB eradication program and individual serum and milk samples were collected immediately before the CITT was performed. In addition, BTM samples from 43 herds (15 high-risk herds and 28 low-risk-herds) included in the study were also collected.

**Table 1:** Number of reactors to the different diagnostic techniques evaluated and apparent prevalence at herd and animal level in total herds and within the groups of the study.

Group	Technique	Herd level		Animal level	
		Positive herds (*n*)	Apparent prevalence (%; Wilson’s 95% CI)	Positive goats (*n*)	Apparent prevalence (%; Wilson’s 95% CI)
High-risk herds	SITT^a^	12/16	75.0% (50.5–89.8)	53/771	6.8% (5.2–8.8)
	CITT^b^	5/16	31.3% (14.2–55.6)	20/771	2.5% (1.6–3.9)
	Serum P22 ELISA^c^	14/16	87.5% (64.0–96.5)	85/771	11.0% (9.0–13.4)
	Individual milk P22 ELISA^c^	13/16	81.3% (57.0–93.4)	116/771	15.0% (12.7–17.7)
	BTM P22 ELISA^c^	5/15	33.3% (15.1–58.2)	-	-
Low-risk herds	SITT	22/37	59.5% (43.5–73.7)	58/1358	4.2% (3.3–5.4)
	CITT	1/37	2.7% (0.5–13.8)	1/1358	0.07% (0.01–0.42)
	Serum P22 ELISA	19/37	51.4% (35.9–66.6)	43/1358	3.1% (2.3–4.2)
	Individual milk P22 ELISA	19/37	51.4% (35.9–66.6)	39/1358	2.8% (2.1–3.9)
	BTM P22 ELISA	0/28	0.0% (0.0–12.0)	–	–
Confirmed TB-infected herds	SITT	6/6	100% (60.9–100)	38/259	14.6% (10.8–19.5)
	CITT	6/6	100% (60.9–100)	21/259	8.1% (5.3–12.0)
	Serum P22 ELISA	6/6	100% (60.9–100)	33/259	12.7% (9.2–17.3)
	Individual milk P22 ELISA	6/6	100% (60.9–100)	58/259	22.3% (17.7–27.8)
	BTM P22 ELISA	2/5	40.0% (11.7–76.9)	-	-

^a^An animal was considered positive to the SITT when there was an increase of ≥ 4 mm in the skin fold thickness and/or the presence of clinical signs was observed. ^b^An animal was considered positive to the CITT when the bovine reaction was greater than the avian reaction by more than 4 mm and/or there were clinical signs at the bovine PPD inoculation site. ^c^An animal was considered positive to P22 ELISA when the E% value was greater than 150.

### 2.2 Serum and milk sample collection

Blood samples were collected by jugular vein puncture using a sterile collection system into tubes with no additives (Vacutainer®, Becton-Dickinson, USA). Then, serum samples were centrifuged (1,500 × g for 10 min), and 1 mL of serum was collected and stored at −20°C, until the ELISA assay was performed. Regarding milk samples, individual milk samples from the animals (4–10 mL) were collected from the mammary gland during the milking session. Milk from BTM was agitated for 5–10 min prior to sample collection (10 mL). Later, individual milk and BTM samples were centrifuged (13,000 × g for 5 min) and 1 mL of whey was collected and stored at −20°C, until the ELISA testing.

### 2.3 Intradermal tuberculin tests

SITT and CITT were performed by the official veterinary services in the framework of the mandatory regional caprine TB program (28), in compliance with Regulation EU 2016/429, Commission Delegated Regulation EU 2020/688 and Spanish Royal Decree 2611/1996. SITT consisted in the intradermal inoculation of 0.1 mL of bovine PPD (CZ Vaccines, Porriño, Spain) on the left-hand side of the neck using a Dermojet syringe (Akra Dermojet, Pau, France). For the CITT, and intradermal inoculation of 0.1 mL of avian PPD on the right side of the neck was also performed. In the case of the SITT, an animal was classified as positive if an increase in the skin fold thickness ≥ 4 mm and/or the presence of clinical signs (oedema, pain, exudation or necrosis) occurred (28). An animal was considered positive to the CITT if the bovine reaction was > 4 mm greater than the avian reaction and/or the presence of clinical signs were observed at the bovine PPD inoculation site (28).

### 2.4 Indirect P22 ELISA

Antibodies against the P22 protein immunocomplex were analysed by employing an in-house indirect ELISA. The P22 ELISA was performed as described previously and using the optimal dilution of serum (1/100) and milk (1/8) samples in 5% skimmed milk/PBS solution (24–26). Afterwards, optical density (OD) was measured at 492 mm with an ELISA reader. Serum, individual milk and BTM samples results were expressed as an ELISA percentage (E%), calculated by employing the following formula E% = [mean sample OD/ (2 x mean of negative control OD)] × 100. Serum, individual milk and BTM samples with E% greater than 150 were considered positive (24, 29).

### 2.5 MTBC and MAP culture

Twenty-one CITT-reactor goats in the study (20 goats belonging to 5 herds from high-risk herds and 1 goat from a low-risk herd) were culled and submitted to *post-mortem* analysis. Lymph nodes from head and thorax and lung samples from 8/21 (38.0%) goats were collected for MTBC culture. Tissue samples were pooled, homogenized, decontaminated using a 0.37% hexadecylpyridinium chloride and cultured on Colestos and 0.2% (w/v) pyruvate-enriched Löwenstein-Jensen media (BioMérieux, Madrid, Spain), as described previously (30). Culture was considered positive when isolates were identified as MTBC by a real-time PCR to detect IS*6110* sequence (31). Finally, the spoligotype and MTBC species of the isolated strains were determined as previously described (32) and spoligotype profiles were assigned using the mycoDB.es spoligotype database (33). Herds with goats that tested culture-positive and were confirmed by PCR were considered TB-infected.

Regarding MAP detection, culture from tissue samples was only performed in a proportion of the herds (5/53; Table 2), being considered a limitation of the study as regards the interpretation of the P22 ELISA results in terms of Sp. In this sense, samples from the ileocecal valve, adjacent tissue and mesenteric lymph nodes were taken for MAP culture as described elsewhere (34). Samples of each animal were pooled, decontaminated using 1.5% HPC (35) and inoculated onto selective media (34). Afterwards, isolates were confirmed by mycobactin-dependency, and specific PCR for detection of IS*900* sequence (36, 37).

**Table 2:** Summary of *ante-mortem* and *post-mortem* techniques performed per herd.

Group	Herd	Goats sampled	*Ante-mortem* diagnosis									*Post-mortem* diagnosis				
			SITT^a^		CITT^b^		Serum P22 ELISA^c^		Milk P22 ELISA^c^		BTM P22 ELISA	Goats culled	MAP PCR-cultured positive	MTBC PCR-cultured positive	Spoligotype	MTBC species
		*n*	*n*	%	*n*	%	*n*	%	*n*	%		*n*	*n*/performed	*n*/performed		
High-risk	1	45	4	8.9%	1	2.2%	10	22.2%	17	37.8%	Positive	1	1/1	1/1	SB 0416	*M. caprae*
	2	57	11	19.3%	10	17.5%	3	5.3%	17	29.8%	Positive	10	3/10	1/1	SB 0157	*M. caprae*
	3	57	15	26.3%	4	7.0%	7	12.3%	7	12.3%	Negative	4	3/4	2/2	SB 0157 (x2)	*M. caprae* (x2)
	4	18	0	0.0%	0	0.0%	1	5.6%	0	0.0%	Negative	–	–	–	–	–
	5	20	0	0.0%	0	0.0%	7	35.0%	7	35.0%	Positive	–	–	–	–	–
	6	57	0	0.0%	0	0.0%	0	0.0%	0	0.0%	Negative	–	–	–	–	–
	7	51	0	0.0%	0	0.0%	0	0.0%	0	0.0%	Negative	–	–	–	–	–
	8	57	1	1.8%	0	0.0%	8	14.0%	9	15.8%	Negative	–	–	–	–	–
	9	57	1	1.8%	0	0.0%	3	5.3%	1	1.8%	–	–	–	–	–	–
	10	57	2	3.5%	0	0.0%	2	3.5%	2	3.5%	Negative	–	–	–	–	–
	11	45	2	4.4%	0	0.0%	1	2.2%	2	4.4%	Negative	–	–	–	–	–
	12	57	3	5.3%	2	3.5%	5	8.8%	4	7.0%	Negative	2	–	2/2	SB 0157 (x2)	*M. caprae* (x2)
	13	56	3	5.4%	0	0.0%	4	7.1%	15	26.8%	Negative	–	–	–	–	–
	14	51	3	5.9%	0	0.0%	12	23.5%	3	5.9%	Positive	–	–	–	–	–
	15	41	4	9.8%	3	7.3%	7	17.1%	12	29.3%	Negative	3	0/2	1/1	SB 0295	*M. bovis*
	16	45	4	8.9%	0	0.0%	15	33.3%	20	44.4%	Positive	–	–	–	–	–
Low-risk	17	26	0	0.0%	0	0.0%	2	7.7%	1	3.8%	Negative	–	–	–	–	–
	18	31	0	0.0%	0	0.0%	0	0.0%	9	29.0%	Negative	–	–	–	–	–
	19	35	0	0.0%	0	0.0%	0	0.0%	0	0.0%	Negative	–	–	–	–	–
	20	40	0	0.0%	0	0.0%	0	0.0%	1	2.5%	Negative	–	–	–	–	–
	21	45	0	0.0%	0	0.0%	0	0.0%	0	0.0%	–	–	–	–	–	–
	22	45	0	0.0%	0	0.0%	0	0.0%	0	0.0%	Negative	–	–	–	–	–
	23	40	0	0.0%	0	0.0%	1	2.5%	1	2.5%	Negative	–	–	–	–	–
	24	9	0	0.0%	0	0.0%	0	0.0%	0	0.0%	–	–	–	–	–	–
	25	40	0	0.0%	0	0.0%	0	0.0%	0	0.0%	Negative	–	–	–	–	–
	26	57	1	1.8%	0	0.0%	0	0.0%	0	0.0%	Negative	–	–	–	–	–
	27	57	1	1.8%	0	0.0%	0	0.0%	0	0.0%	Negative	–	–	–	–	–
	28	57	1	1.8%	0	0.0%	1	1.8%	1	1.8%	Negative	–	–	–	–	–
	29	57	1	1.8%	0	0.0%	3	5.3%	0	0.0%	Negative	–	–	–	–	–
	30	51	1	2.0%	0	0.0%	0	0.0%	0	0.0%	Negative	–	–	–	–	–
	31	57	2	3.5%	0	0.0%	10	17.5%	7	12.3%	Negative	–	–	–	–	–
	32	26	1	3.8%	0	0.0%	1	3.8%	1	3.8%	–	–	–	–	–	–
	33	45	2	4.4%	0	0.0%	1	2.2%	0	0.0%	Negative	–	–	–	–	–
	34	35	2	5.7%	0	0.0%	3	8.6%	2	5.7%	Negative	–	–	–	–	–
	35	50	4	8.0%	0	0.0%	4	8.0%	2	4.0%	Negative	–	–	–	–	–
	36	51	16	31.4%	0	0.0%	4	7.8%	3	5.9%	Negative	–	–	–	–	–
	37	11	0	0.0%	0	0.0%	0	0.0%	1	9.1%	–	–	–	–	–	–
	38	51	0	0.0%	0	0.0%	0	0.0%	0	0.0%	Negative	–	–	–	–	–
	39	10	0	0.0%	0	0.0%	1	10.0%	1	10.0%	–	–	–	–	–	–
	40	31	0	0.0%	0	0.0%	1	3.2%	1	3.2%	Negative	–	–	–	–	–
	41	20	0	0.0%	0	0.0%	3	15.0%	3	15.0%	Negative	–	–	–	–	–
	42	4	0	0.0%	0	0.0%	0	0.0%	0	0.0%	–	–	–	–	–	–
	43	57	1	1.8%	0	0.0%	0	0.0%	1	1.8%	Negative	–	–	–	–	–
	44	45	1	2.2%	0	0.0%	1	2.2%	1	2.2%	Negative	–	–	–	–	–
	45	35	1	2.9%	0	0.0%	0	0.0%	1	2.9%	Negative	–	–	–	–	–
	46	51	2	3.9%	0	0.0%	0	0.0%	0	0.0%	Negative	–	–	–	–	–
	47	23	1	4.3%	0	0.0%	0	0.0%	0	0.0%	Negative	–	–	–	–	–
	48	21	2	9.5%	0	0.0%	0	0.0%	0	0.0%	Negative	–	–	–	–	–
	49	31	2	6.5%	0	0.0%	1	3.2%	0	0.0%	–	–	–	–	–	–
	50	51	6	11.8%	0	0.0%	3	5.9%	0	0.0%	Negative	–	–	–	–	–
	51	57	8	14.0%	0	0.0%	1	1.8%	1	1.8%	Negative	–	–	–	–	–
	52	4	1	25.0%	0	0.0%	1	25.0%	0	0.0%	–	–	–	–	–	–
	53	2	1	50.0%	1	50.0%	1	50.0%	1	50.0%	–	1	1/1	1/1	SB 0157	*M. caprae*

^a^An animal was considered positive to the SITT when there was an increase of ≥ 4 mm in the skin fold thickness and/or the presence of clinical signs was observed. ^b^An animal was considered positive to the CITT when the bovine reaction was greater than the avian reaction by more than 4 mm and/or there were clinical signs at the bovine PPD inoculation site. ^c^An animal was considered positive to P22 ELISA when the E% value was greater than 150.

### 2.6 Statistical analysis

The proportion of reactor animals along with the Wilson 95% confidence interval (95% CI) was calculated using WinPepi version 11.65 (38). The prevalence of positive goats based on the different techniques was compared using the McNemar’s test. Agreement between tests was measured with the kappa statistic (k) and interpreted as follows: <0.000 no agreement, 0.000–0.200 slight, 0.201–0.400 fair, 0.401–0.600 moderate, 0.601–0.800 substantial and 0.801–1.000 almost perfect agreement (39). Spearman’s rank correlation coefficient (r_s_) was employed to assess the relation between E% values in serum and individual milk samples in the P22 ELISA and between the mean E% values obtained in the individual milk P22 ELISA and E% values obtained in BTM samples.

## 3 Results

### 3.1 High-risk herds (*n* = 16)

The number and percentage of reactors to SITT, CITT and P22 ELISA using individual serum and individual milk and BTM in herds included in the study are summarized in Tables 1, 2. Out of the 16 high-risk herds, SITT and CITT reactors were found in 12 (75.0%) and 5 (31.3%) of them respectively, while one or more P22 ELISA-positive animals were found in 14 (87.5%), 13 (81.3%) and 5 (33.3%) herds using serum, individual milk and BTM samples, respectively.

Individual apparent prevalence obtained using the P22 ELISA in serum (11.0%) and especially individual milk (15.0%) was higher than using SITT (6.8%) and CITT (2.5%), with also a significantly higher (*p* < 0.001) proportion of reactors found in the P22 ELISA when considering the individual milk samples compared with serum (Table 1; Supplementary Figure 1). Agreement between techniques is detailed in Table 3. Agreement between cellular and humoral techniques was slight, while it was from moderate to substantial when comparison was made only considering cellular or humoral tests separately. A positive correlation (r_s_ = 0.620; *p* < 0.001) (Supplementary Figure 2) was observed between E% values in serum and individual milk, which was stronger (r_s_ = 0.789; *p* < 0.001) when considering E% values in BTM and the mean E% values in individual milk samples from a given herd. In addition, among the positive BTM samples, the lowest herd prevalence based on individual milk was 5.9%.

**Table 3:** Agreement (kappa value) between the different diagnostic techniques evaluated at the animal level.

Technique		High-risk herds (*n* = 771 goats)	Low-risk herds (*n* = 1,358 goats)	Confirmed TB-infected herds (*n* = 259 goats)
SITT vs.	CITT	0.530	0.032	0.678
	Serum P22 ELISA	0.145	0.106	0.168
	Individual milk P22 ELISA	0.157	0.071	0.139
CITT vs.	Serum P22 ELISA	0.115	0.044	0.260
	Individual milk P22 ELISA	0.123	0.049	0.209
Serum P22 ELISA vs.	Individual milk P22 ELISA	0.607	0.522	0.567

### 3.2 Low-risk herds (*n* = 37)

In the low-risk herds, a lower reactivity at herd and individual level was observed when performing cell-based and humoral techniques compared to high-risk herds (Table 1). Within low-risk herds, at least one reactor was found in 59.5 and 2.7% of the herds when performing the SITT and CITT, respectively. Regarding P22 ELISA, 51.4% of the low-risk herds contained reactors to both serum and individual milk samples but none of the herds were positive in the BTM testing (Table 1).

Similarly to the herd level, the percentage of reactors in the P22 ELISA using serum (3.1%) and milk (2.8%) samples were again lower compared to SITT (4.2%), but higher than when using CITT (0.07%). Also, in this set, a moderate agreement was observed between the P22 ELISA in serum and milk (Table 3). However, a slight agreement was observed between the SITT and CITT specifically and between cell-based and humoral-based techniques in general. Moreover, a positive but weaker correlation (r_s_ = 0.363; *p* < 0.001) (Supplementary Figure 2) than that observed in the high-risk herds, was found between E% values in serum and individual milk.

### 3.3 Infected herds confirmed by bacteriology (*n* = 6)

MTBC infection was confirmed in six herds (five high-risk ones and one low-risk herd). Three spoligotypes were found: SB0157 (*M. caprae*; in four herds) and SB0416 (*M. caprae*), and SB0295 (*M. bovis*) in one each herd. In addition, PTB infection was confirmed by bacteriological culture in four of the five TB-infected herds for which PTB data were available (Table 2).

Reactivity among the confirmed TB-infected herds is summarized in Table 1. Reactors in the SITT, CITT and P22 ELISA using serum and individual milk samples were found in all six TB-infected herds, while only 2/5 were detected using BTM samples. Similarly to what was observed in high-risk herds, a higher proportion of goats were detected using milk based P22 ELISA (22.3%) compared to serum (12.7%), SITT (14.6%) and CITT (8.1%) (Table 1), with a significantly higher (*p* < 0.001) proportion of reactors found in the P22 ELISA in the individual milk samples compared with serum. In addition, a moderate to substantial agreement was observed among cellular and humoral techniques, but it was only from slight to fair when comparing the intradermal tests versus the P22 ELISA (Table 3). Moreover, a strong correlation (r_s_ = 0.696; *p* < 0.001) (Supplementary Figure 2) was observed between E% values in serum and individual milk but only a weak not statistically significant correlation (r_s_ = 0.300; *p* > 0.05) was found between E% values in BTM and the mean E% values in individual milk samples.

## 4 Discussion

A critical aspect for the TB persistence is the epidemiological role that goats play in the maintenance of the infection, not only being susceptible to MTBC infection but also acting as a reservoir (6, 7, 40). The challenging diagnosis of TB in goats and the development of humoral-based methodologies, including the P22 ELISA, over the last few years highlight the need for a large-scale study in order to evaluate the usefulness of this technique within a TB eradication program in goats, which was the main objective of the present study.

Regarding P22 ELISA results in high-risk herds, a higher reactivity was observed at herd and animal level when using serum and individual milk samples compared the intradermal tests, thus suggesting a higher Se of the P22 ELISA given the known limitations of intradermal tests (14, 15). In addition, even though the proportion of positive herds was similar both when considering serum and individual milk samples, at the individual level a significantly higher proportion of goats were found in the individual milk compared with serum samples, what could indicate a higher Se of the test when using the former. This trend was also observed in the six herds with a confirmed TB-infection by culture. This findings align with a previous study (25) evaluating the reactivity of the P22 ELISA in serum and individual milk samples at a different sampling times in a caprine TB-infected herd that described a slightly higher Se of the P22 ELISA in individual milk samples. Nonetheless, another study (24) reported a slightly lower Se of the P22 ELISA when using individual milk samples compared to serum in goats with confirmed TB-infection by culture and/or the presence of TB-like lesions (TBLLs). However, since these studies were based on a small sample size (*n* < 150 animals), our larger dataset may provide more representative results when applying the P22 ELISA at a larger scale. Therefore, milk-based testing may prove effective in identifying a higher number of reactors and can be an excellent alternative for TB diagnosis due to the rapid and non-invasive sampling of the goats during the milking session by the farm-operators, leading to fewer sampling costs-associated and animal welfare consequences compared to serum (24). These findings along with the low agreement between the P22 ELISA and intradermal tests in observed in the study highlight the potential use of the P22 ELISA as a complementary technique for surveillance of TB-infected herds in the context of an eradication program.

Regarding BTM samples, in our study a lower proportion of herds were considered positive when using BTM compared with individual samples both among the high-risk herds and the TB-confirmed herds (between 33.3 and 40.0% depending on the study group). This suggests a lower Se of this diagnostic strategy compared with a previous study from Waters and collaborators (41) evaluating BTM-based sampling in cattle herds, that reported a Se of 82.3%. However, differences in BTM performance may be influenced by the humoral technique used, species-specific responses (22, 26) or a dilution effect of the samples (24, 42, 43) since BTM samples in the study from Waters and collaborators were collected from herds with an unclear prevalence rate of TB (41). In this sense, a strong correlation was observed in our study between E% values in BTM and the mean E% values in individual milk samples within herds.

In the low-risk herds, herd and animal-level prevalence were similar in serum and individual milk P22 ELISA and SITT, but higher than CITT. It is widely known that the Sp of the SITT may be compromised under certain circumstances, such as the infection with non-tuberculous mycobacteria (NTM) (44), or infection with/vaccination against MAP (11, 16). For this reason, regional caprine TB programs in Spain allow the use of the CITT to differentiate between TB-infected animals from those sensitized to bovine PPD as a result of exposure to NTM or vaccination against PTB (12, 14, 45, 46). In general terms, PTB vaccination is widely used in caprine herds in Spain (5). Nevertheless, the absence of an official PTB eradication program and an associated vaccination registry, it was not possible to know which herds were vaccinated in this study. The detection of TB infection by culture in a low-risk herd (in which SITT and CITT detected) highlights the risk of infection also in this low-risk category. Therefore, it is difficult to determine if the higher proportion of reactors found in the P22 ELISA in the low-risk herds is related to a lack of Sp or an increased Se. A previous study (18) reported that the Sp of P22 ELISA may be significantly affected in MAP-vaccinated and TB-free goats where compulsory intradermal tuberculin tests were also performed (conditions that may commonly occur in the context of an eradication program in goats), reaching values of nearly 40% 12 months post-vaccination. In addition, Infantes-Lorenzo and collaborators (29) observed that Sp of P22 ELISA may drop to 56.1% in non-vaccinated herds against PTB, probably due to the high prevalence of MAP in goats in certain countries including Spain (19). In this sense, it is possible that infection with MAP or other NTMs (since in our study PTB infection was confirmed in 4/5 of the herds from which *post-mortem* data could be obtained) as well as MAP-vaccination of the animals could have compromised the Sp of the P22 ELISA and led to the detection of false-positive reactors not only in low-risk herds but also in high-risk and confirmed TB-infected herds. However, though a similar reactivity was observed in individual milk and serum in low-risk herds, the higher rate of positive animals in the P22 ELISA using individual milk compared to serum and intradermal test in high-risk and TB-infected herds tests observed in the present study suggest that individual milk samples may be a valuable sample for TB diagnosis. Furthermore, a more stringent cut-off used in previous studies (21, 22, 29) was also evaluated, reporting a higher reactivity in high-risk and confirmed TB-infected herds (especially using individual milk samples). Nevertheless, it was not finally considered due to the significant loss of Sp (mainly in serum) in low-risk herds (data not shown).

Moreover, P22 ELISA in BTM samples yielded the lowest number of positives, suggesting it to be the most specific technique among the evaluated in the present study. In line with this findings, Waters and collaborators (41) observed that none of the 185 TB-free cattle herds reacted to a commercial ELISA technique detecting antibodies against *M. bovis* in BTM samples. However, although the high Sp of P22 ELISA in BTM samples may be related to a limited Se in TB-infected herds, in our study the P22 ELISA in BTM detected 40.0% of the herds with confirmed TB-infection. This high Sp and acceptable Se of this type of sample may indicate the need for adjustment of the optimal cut-off, which should be addressed in further studies with a higher sample size assessing different interpretation criteria and considering specific limitations of BTM samples such as the dilution effect (24, 42, 43).

## 5 Conclusion

In conclusion, the P22 ELISA in serum and individual milk samples shows potential as a complementary tool to enhance the Se of the intradermal test and accelerate eradication efforts. In addition, while serum and individual milk samples showed similar reactivity in low-risk herds the latter would be valuable for maximizing the number of reactors using the P22 ELISA in infected settings.

## Data Availability

The raw data supporting the conclusions of this article will be made available by the authors, without undue reservation.

## References

[1] Pesciaroli M Alvarez J Boniotti MB Cagiola M Di Marco V Marianelli C. Tuberculosis in domestic animal species. Res Vet Sci. (2014) 97:S78–85. doi: 10.1016/j.rvsc.2014.05.015, PMID: 25151859

[2] Gortazar C Vicente J Boadella M Ballesteros C Galindo RC Garrido J. Progress in the control of bovine tuberculosis in spanish wildlife. Vet Microbiol. (2011) 151:170–8. doi: 10.1016/j.vetmic.2011.02.041, PMID: 21440387

[3] Olea-Popelka F Muwonge A Perera A Dean AS Mumford E Erlacher-Vindel E. Zoonotic tuberculosis in human beings caused by *Mycobacterium bovis*-a call for action. Lancet Infect Dis. (2017) 17:e21–5. doi: 10.1016/S1473-3099(16)30139-6, PMID: 27697390

[4] Vayr F Martin-Blondel G Savall F Soulat JM Deffontaines G Herin F. Occupational exposure to human *Mycobacterium bovis* infection: a systematic review. PLoS Negl Trop Dis. (2018) 12:e0006208. doi: 10.1371/journal.pntd.0006208, PMID: 29337996 PMC5786333

[5] Juste RA Perez V. Control of paratuberculosis in sheep and goats. Vet Clin N Am Food Anim Pract. (2011) 27:127–38. doi: 10.1016/j.cvfa.2010.10.020, PMID: 21215897

[6] Napp S Allepuz A Mercader I Nofrarías M López-Soria S Domingo M. Evidence of goats acting as domestic reservoirs of bovine tuberculosis. Vet Rec. (2013) 172:663–3. doi: 10.1136/vr.101347, PMID: 23687108

[7] Rodríguez S Bezos J Romero B De Juan L Álvarez J Castellanos E. *Mycobacterium caprae* infection in livestock and wildlife. Spain Emerg Infect Dis. (2011) 17:532–5. doi: 10.3201/eid1703.100618, PMID: 21392452 PMC3165998

[8] Martínez-Lirola M Herranz M Buenestado Serrano S Rodríguez-Grande C Dominguez Inarra E Garrido-Cárdenas JA. A one health approach revealed the long-term role of *Mycobacterium caprae* as the hidden cause of human tuberculosis in a region of Spain, 2003 to 2022. Eur Secur. (2023) 28:852. doi: 10.2807/1560-7917.ES.2023.28.12.2200852, PMID: 36951787 PMC10037661

[9] Eurostat. (2024). Goats population - anual data. Available online at: https://ec.europa.eu/eurostat/databrowser/view/APRO_MT_LSGOAT/default/table?lang=en (accesed April 2, 2025).

[10] Ministerio de Agricultura, Pesca Alimentación. (2023). El sector ovino y caprino de carne en cifras. Principales indicadores económicos. Available online at: https://www.mapa.gob.es/es/ganaderia/estadisticas/indicadoreseconomicosdelsectorovinoycaprino_carne_2024sagosto_tcm30-511496.pdf (accesed April 2, 2025).

[11] Ministerio de Agricultura, Pesca Alimentación. (2025). Programa Nacional de Erradicación de Tuberculosis Bovina 2025 (Infección por el complejo Mycobacterium tuberculosis). Available online at: https://www.mapa.gob.es/es/ganaderia/temas/sanidad-animal-higiene-ganadera/programatb2025_18022025_tcm30-698268.pdf (accesed April 2, 2025).

[12] Ministerio de Agricultura, Pesca Alimentación. (2024). Manual para el control de la infección por el CMT en establecimientos de ganado caprino incluidos en el programa nacional de erradicación de la infección por el complejo Mycobacterium tuberculosis (CMT). Available online at: https://www.mapa.gob.es/en/ganaderia/temas/sanidad-animal-higiene-ganadera/8manualcaprino2024_tcm38-553693.pdf (accesed April 2, 2025).

[13] Espinosa J Fernández M Royo M Grau A Ángel Collazos J Benavides J. Influence of vaccination against paratuberculosis on the diagnosis of caprine tuberculosis during official eradication programmes in Castilla y León (Spain). Transbounding Emerging Dis. (2021) 68:692–703. doi: 10.1111/tbed.13732, PMID: 32668068

[14] Bezos J Marqués S Álvarez J Casal C Romero B Grau A. Evaluation of single and comparative intradermal tuberculin tests for tuberculosis eradication in caprine flocks in Castilla y León (Spain). Res Vet Sci. (2014) 96:39–46. doi: 10.1016/j.rvsc.2013.10.007, PMID: 24239314

[15] Roy A Infantes-Lorenzo JA De La Cruz ML Domínguez L Álvarez J Bezos J. Accuracy of tuberculosis diagnostic tests in small ruminants: a systematic review and meta-analysis. Prev Vet Med. (2020) 182:105102. doi: 10.1016/j.prevetmed.2020.105102, PMID: 32739695

[16] Álvarez J De Juan L Bezos J Romero B Sáez JL Gordejo FJR. Interference of paratuberculosis with the diagnosis of tuberculosis in a goat flock with a natural mixed infection. Vet Microbiol. (2008) 128:72–80. doi: 10.1016/j.vetmic.2007.08.034, PMID: 17954015

[17] Bezos J Álvarez J Romero B Aranaz A Juan LD. Tuberculosis in goats: assessment of current in vivo cell-mediated and antibody-based diagnostic assays. Vet J. (2012) 191:161–5. doi: 10.1016/j.tvjl.2011.02.010, PMID: 21388843

[18] Roy Á Infantes-Lorenzo JA Blázquez JC Venteo Á Mayoral FJ Domínguez M. Temporal analysis of the interference caused by paratuberculosis vaccination on the tuberculosis diagnostic tests in goats. Prev Vet Med. (2018) 156:68–75. doi: 10.1016/j.prevetmed.2018.05.010, PMID: 29891147

[19] Jiménez-Martín D García-Bocanegra I Risalde MA Fernández-Molera V Jiménez-Ruiz S Isla J. Epidemiology of paratuberculosis in sheep and goats in southern Spain. Prev Vet Med. (2022) 202:105637. doi: 10.1016/j.prevetmed.2022.105637, PMID: 35378433

[20] Pérez De Val B Nofrarías M López-Soria S Garrido JM Vordermeier H Villarreal-Ramos B. Effects of vaccination against paratuberculosis on tuberculosis in goats: diagnostic interferences and cross-protection. BMC Vet Res. (2012) 8:191. doi: 10.1186/1746-6148-8-191, PMID: 23072619 PMC3514378

[21] Arrieta-Villegas C Infantes-Lorenzo JA Bezos J Grasa M Vidal E Mercader I. Evaluation of P22 antigenic complex for the immuno-diagnosis of tuberculosis in BCG vaccinated and unvaccinated goats. Front Vet Sci. (2020) 7:374. doi: 10.3389/fvets.2020.00374, PMID: 32714950 PMC7351524

[22] Bezos J Roy Á Infantes-Lorenzo JA González I Venteo Á Romero B. The use of serological tests in combination with the intradermal tuberculin test maximizes the detection of tuberculosis infected goats. Vet Immunol Immunopathol. (2018) 199:43–52. doi: 10.1016/j.vetimm.2018.03.006, PMID: 29678229

[23] Infantes-Lorenzo JA Moreno I Risalde MDLÁ Roy Á Villar M Romero B. Proteomic characterisation of bovine and avian purified protein derivatives and identification of specific antigens for serodiagnosis of bovine tuberculosis. Clin Proteomics. (2017) 14:36. doi: 10.1186/s12014-017-9171-z, PMID: 29142508 PMC5669029

[24] Roy A Infantes-Lorenzo JA Domínguez M Moreno I Pérez M García N. Evaluation of a new enzyme-linked immunosorbent assay for the diagnosis of tuberculosis in goat milk. Res Vet Sci. (2020) 128:217–23. doi: 10.1016/j.rvsc.2019.12.009, PMID: 31835123

[25] Ortega J Infantes-Lorenzo JA Roy A De Juan L Romero B Moreno I. Factors affecting the performance of P22 ELISA for the diagnosis of caprine tuberculosis in milk samples. Res Vet Sci. (2022) 145:40–5. doi: 10.1016/j.rvsc.2022.02.008, PMID: 35151157

[26] Casal C Infantes JA Risalde MA Díez-Guerrier A Domínguez M Moreno I. Antibody detection tests improve the sensitivity of tuberculosis diagnosis in cattle. Res Vet Sci. (2017) 112:214–21. doi: 10.1016/j.rvsc.2017.05.012, PMID: 28521256

[27] Jiménez-Martín D García-Bocanegra I Risalde MA Napp S Domínguez M Romero B. *Mycobacterium tuberculosis* complex in domestic goats in southern Spain. Prev Vet Med. (2024) 227:106204. doi: 10.1016/j.prevetmed.2024.106204, PMID: 38604014

[28] Orden de 2 de Octubre de 2017. (2017). Diario oficial de Extremadura. Available online at: https://doe.juntaex.es/eli/es-ex/o/2017/10/02/(1)/dof/spa/pdf (accesed April 2, 2025).

[29] Infantes-Lorenzo JA Moreno I Roy A Risalde MA Balseiro A De Juan L. Specificity of serological test for detection of tuberculosis in cattle, goats, sheep and pigs under different epidemiological situations. BMC Vet Res. (2019) 15:70. doi: 10.1186/s12917-019-1814-z, PMID: 30823881 PMC6397464

[30] Corner LAL Gormley E Pfeiffer DU. Primary isolation of *Mycobacterium bovis* from bovine tissues: conditions for maximising the number of positive cultures. Vet Microbiol. (2012) 156:162–71. doi: 10.1016/j.vetmic.2011.10.016, PMID: 22074859

[31] Michelet L De Cruz K Karoui C Tambosco J Moyen JL Hénault S. Second line molecular diagnosis for bovine tuberculosis to improve diagnostic schemes. PLoS One. (2018) 13:e0207614. doi: 10.1371/journal.pone.0207614, PMID: 30475835 PMC6261039

[32] Aranaz A Liébana E Mateos A Dominguez L Vidal D Domingo M. Spacer oligonucleotide typing of *Mycobacterium bovis* strains from cattle and other animals: a tool for studying epidemiology of tuberculosis. J Clin Microbiol. (1996) 34:2734–40. doi: 10.1128/jcm.34.11.2734-2740.1996, PMID: 8897175 PMC229396

[33] Rodriguez-Campos S González S De Juan L Romero B Bezos J Casal C. A database for animal tuberculosis (mycoDB.Es) within the context of the spanish national programme for eradication of bovine tuberculosis. Infect Genet Evol. (2012) 12:877–82. doi: 10.1016/j.meegid.2011.10.008, PMID: 22027158

[34] de Juan L Álvarez J Romero B Bezos J Castellanos E Aranaz A. Comparison of four different culture media for isolation and growth of type II and type I/III *Mycobacterium avium* subsp. paratuberculosis strains isolated from cattle and goats. Appl Environ Microbiol. (2006) 72:5927–32. doi: 10.1128/AEM.00451-06, PMID: 16957212 PMC1563672

[35] Greig A Stevenson K Henderson D Perez V Hughes V Pavlik I. Epidemiological study of paratuberculosis in wild rabbits in Scotland. J Clin Microbiol. (1999) 37:1746–51. doi: 10.1128/JCM.37.6.1746-1751.1999, PMID: 10325318 PMC84940

[36] Castellanos E Aranaz A De Juan L Álvarez J Rodríguez S Romero B. Single nucleotide polymorphisms in the IS 900 sequence of *Mycobacterium avium* subsp. paratuberculosis are strain type specific. J Clin Microbiol. (2009) 47:2260–4. doi: 10.1128/JCM.00544-09, PMID: 19439536 PMC2708469

[37] Coetsier C Vannuffel P Blondeel N Denef JF Cocito C Gala JL. Duplex PCR for differential identification of *Mycobacterium bovis*, M. Avium, and *M. avium* subsp. paratuberculosis in formalin-fixed paraffin-embedded tissues from cattle. J Clin Microbiol. (2000) 38:3048–54. doi: 10.1128/JCM.38.8.3048-3054.2000, PMID: 10921976 PMC87183

[38] Abramson JH. WINPEPI updated: computer programs for epidemiologists, and their teaching potential. Epidemiol Perspect Innov. (2011) 8:1. doi: 10.1186/1742-5573-8-1, PMID: 21288353 PMC3041648

[39] Landis JR Koch GG. The measurement of observer agreement for categorical data. Biometrics. (1977) 33:159. doi: 10.2307/2529310, PMID: 843571

[40] Pérez De Val B López-Soria S Nofrarías M Martín M Vordermeier HM Villarreal-Ramos B. Experimental model of tuberculosis in the domestic goat after endobronchial infection with *Mycobacterium caprae*. Clin Vaccine Immunol. (2011) 18:1872–81. doi: 10.1128/CVI.05323-11, PMID: 21880849 PMC3209027

[41] Waters WR Buddle BM Vordermeier HM Gormley E Palmer MV Thacker TC. Development and evaluation of an enzyme-linked immunosorbent assay for use in the detection of bovine tuberculosis in cattle. Clin Vaccine Immunol. (2011) 18:1882–8. doi: 10.1128/CVI.05343-11, PMID: 21918115 PMC3209037

[42] Buddle BM Wilson T Luo D Voges H Linscott R Martel E. Evaluation of a commercial enzyme-linked immunosorbent assay for the diagnosis of bovine tuberculosis from milk samples from dairy cows. Clin Vaccine Immunol. (2013) 20:1812–6. doi: 10.1128/CVI.00538-13, PMID: 24132605 PMC3889516

[43] Lombard JE Byrem TM Wagner BA McCluskey BJ. Comparison of milk and serum enzyme-linked immunosorbent assays for diagnosis of *Mycobacterium avium* subspecies paratuberculosis infection in dairy cattle. J Vet Diagn Invest. (2006) 18:448–58. doi: 10.1177/104063870601800504, PMID: 17037612

[44] Gomez-Buendia A Ortega J Diez-Guerrier A Rendahl A Saez JL Bezos J. Evaluating the ability of non-tuberculous mycobacteria to induce non-specific reactions in bovine tuberculosis diagnostic tests in guinea pigs and cattle. Vet Microbiol. (2024) 298:110250. doi: 10.1016/j.vetmic.2024.110250, PMID: 39265280

[45] Fernández-Veiga L Fuertes M Geijo MV Pérez De Val B Vidal E Michelet L. Differences in skin test reactions to official and defined antigens in guinea pigs exposed to non-tuberculous and tuberculous bacteria. Sci Rep. (2023) 13:2936. doi: 10.1038/s41598-023-30147-4, PMID: 36806813 PMC9941491

[46] EFSA Panel on Animal Health and Welfare (AHAW) More S Bøtner A Butterworth A Calistri P Depner K. Assessment of listing and categorisation of animal diseases within the framework of the animal health law (regulation (EU) no 2016/429): paratuberculosis. EFSA J. (2017) 15:e4960. doi: 10.2903/j.efsa.2017.4960, PMC7010113 32625604

