# Supplementary figures and images for: Field evaluation of the P22 ELISA for diagnosis of caprine tuberculosis in an endemic area
Source: Front Vet Sci. 2025 Jul 24;12:1628812. doi: 10.3389/fvets.2025.1628812 (PMC12329796; doi:10.3389/fvets.2025.1628812)

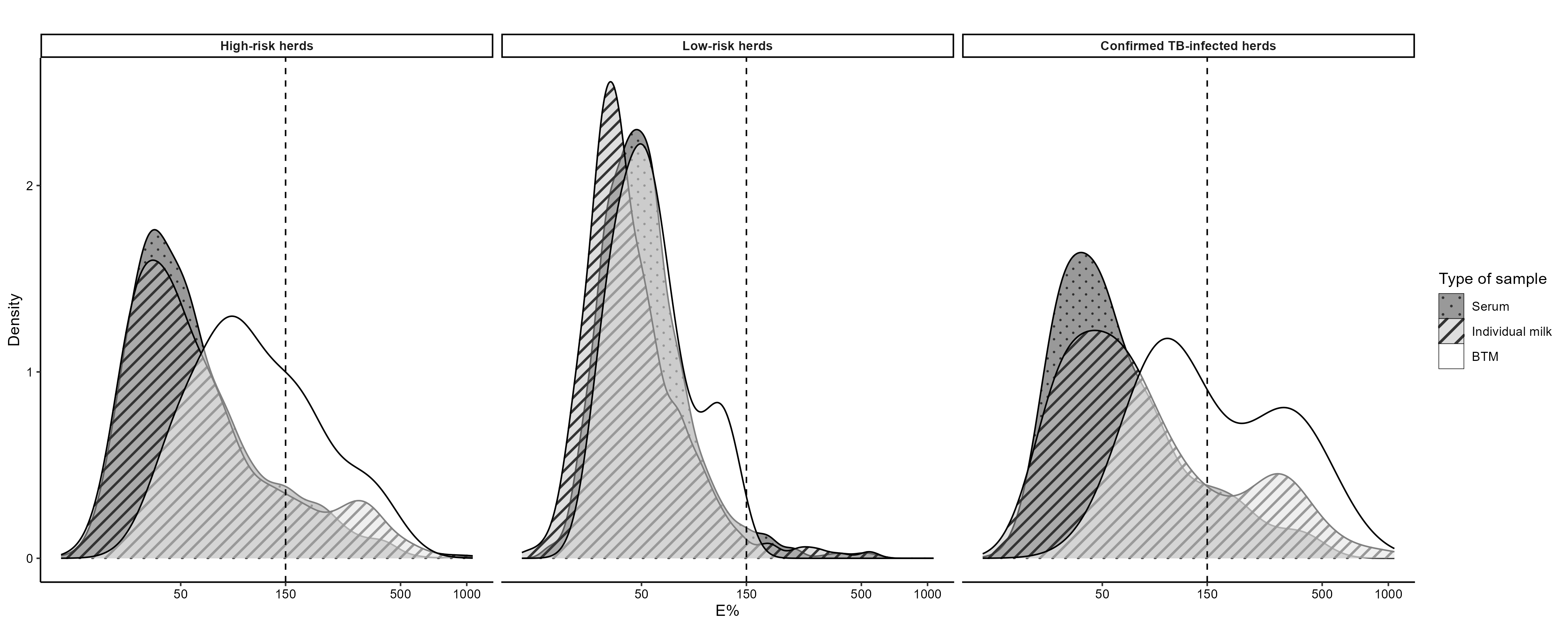

Supplement: Supplementary file 1 [file Image_1.TIFF]

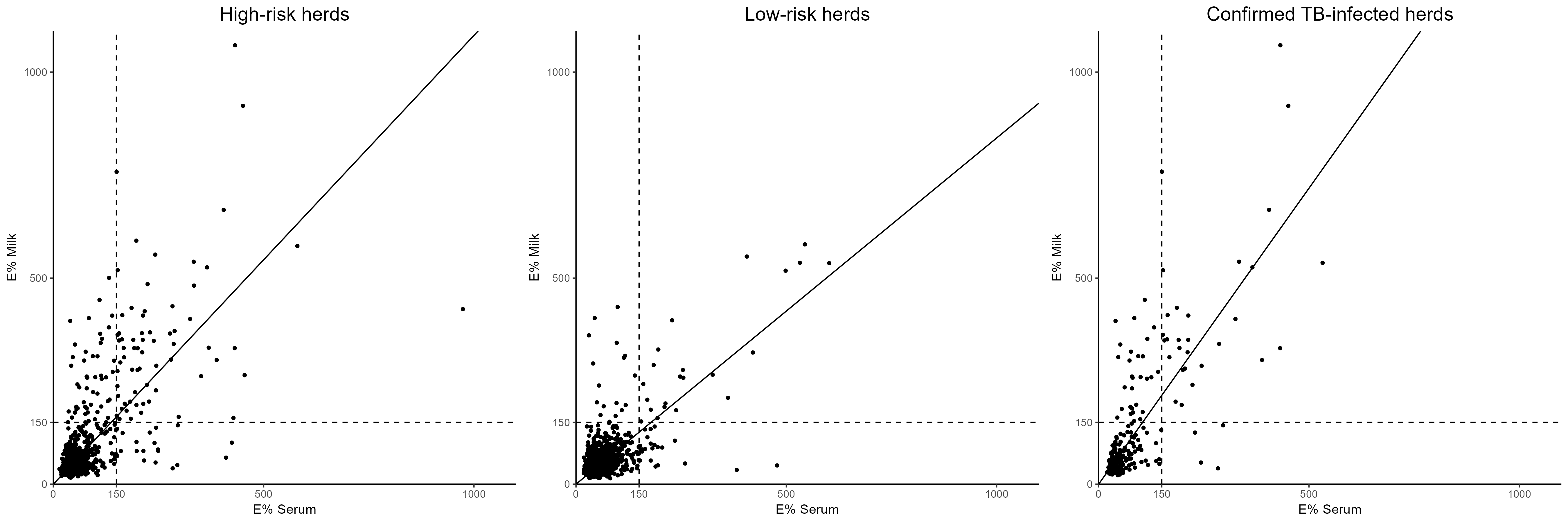

Supplement: Supplementary file 2 [file Image_2.TIFF]
