# Supplementary material for: Field evaluation of the P22 ELISA for diagnosis of caprine tuberculosis in an endemic area
Source: Front Vet Sci. 2025 Jul 24;12:1628812. doi: 10.3389/fvets.2025.1628812 (PMC12329796; doi:10.3389/fvets.2025.1628812)
Supplement: Supplementary file 3 [file Table_1.DOCX]

Supplementary Material

# Supplementary Table

## Table 1. Scale of sample size taken as a function of herd size

| Number of animals within herd | Sample size |
| --- | --- |
| 1-25 | All |
| 26-30 | 26 |
| 31-40 | 31 |
| 41-50 | 35 |
| 51-70 | 40 |
| 71-100 | 45 |
| 101-200 | 50 |
| 201-1200 | 57 |
| >1200 | 59 |

2**. Supplementary figures**

**2.1 Density distribution of E% values for serum, individual milk and bulk tank milk samples by herd type. Dashed lines represent the 150 E% cut-off.**

**2.2 Correlation of E% values between serum and individual milk samples by herd type. Dashed lines represent the 150 E% cut-off.**
